# Soluble CD5 and CD6: Lymphocytic Class I Scavenger Receptors as Immunotherapeutic Agents

**DOI:** 10.3390/cells9122589

**Published:** 2020-12-03

**Authors:** María Velasco-de Andrés, Sergi Casadó-Llombart, Cristina Català, Alejandra Leyton-Pereira, Francisco Lozano, Fernando Aranda

**Affiliations:** 1 Immunoreceptors del Sistema Innat i Adaptatiu, Institut d’Investigacions Biomèdiques August Pi i Sunyer, 08036 Barcelona, Spain; mvelascod@clinic.cat (M.V.-d.A.); secasado@clinic.cat (S.C.-L.); catala@clinic.cat (C.C.); leyton@clinic.cat (A.L.-P.); 2 Servei d’Immunologia, Hospital Clínic de Barcelona, 08036 Barcelona, Spain; 3 Immunoregulació de la Resposta Innata i Adaptativa, Department de Biomedicina, Universitat de Barcelona, 08036 Barcelona, Spain; 4 Program of Immunology and Immunotherapy, Center for Applied Medical Research (CIMA), University of Navarra, 31008 Pamplona, Spain; 5 Instituto de Investigación de Navarra (IDISNA), 31008 Pamplona, Spain

**Keywords:** soluble CD5, soluble CD6, immunomodulation, immunotherapy, infection, cancer, autoimmunity

## Abstract

CD5 and CD6 are closely related signal-transducing class I scavenger receptors mainly expressed on lymphocytes. Both receptors are involved in the modulation of the activation and differentiation cell processes triggered by clonotypic antigen-specific receptors present on T and B cells (TCR and BCR, respectively). To serve such a relevant immunomodulatory function, the extracellular region of CD5 and CD6 interacts with soluble and/or cell-bound endogenous counterreceptors but also microbial-associated molecular patterns (MAMPs). Evidence from genetically-modified mouse models indicates that the absence or blockade of CD5- and CD6-mediated signals results in dysregulated immune responses, which may be deleterious or advantageous in some pathological conditions, such as infection, cancer or autoimmunity. Bench to bedside translation from transgenic data is constrained by ethical concerns which can be overcome by exogenous administration of soluble proteins acting as decoy receptors and leading to transient “functional knockdown”. This review gathers information currently available on the therapeutic efficacy of soluble CD5 and CD6 receptor infusion in different experimental models of disease. The existing proof-of-concept warrants the interest of soluble CD5 and CD6 as safe and efficient immunotherapeutic agents in diverse and relevant pathological conditions.

## 1. Introduction

Scavenger Receptors (SRs) comprise a structurally diverse superfamily of proteins involved in a wide range of biological functions through the recognition of a variety of endogenous and exogenous structures [1,2]. SRs are mainly expressed by cells from epithelial barriers and the innate immune system, but T and B lymphocytes, the prototypical cell type of the adaptive immune system, are also equipped with some representatives of the SR superfamily. This is the case of CD5 and CD6 receptors, two members of the class I type SRs, defined by the presence of several scavenger receptor cysteine-rich (SRCR) domain repeats [3].

The CD5 and CD6 lymphocyte receptors share a high degree of functional and structural homology, reflecting their origin from duplication of a common ancestral gene located at homologous regions of human and mouse chromosomes 11 and 9, respectively [1]. Both are integral trans-membrane glycoproteins, characterized by three extracellular SRCR domains and a cytoplasmic tail adapted for intracellular signal transduction, in spite of lacking intrinsic catalytic activity [4]. Importantly, CD5 and CD6 are co-receptors physically associated with each other [5], as well as with the clonotypic antigen-specific receptor complex of T (TCR) and B1a (BCR) cells [6,7,8], enabling modulation and fine-tuning of the activation/death signals delivered upon specific antigen recognition [9]. This confers both receptors important immunomodulatory properties as bona fide immune checkpoint regulators [9,10]. Additionally, accumulating evidence shows that CD5 and CD6 are pattern recognition receptors (PRRs) that also sense the presence of microbial-associated molecular patterns (MAMPs) from different origins (bacterial, fungal, viral or parasitic) [11,12,13,14]. The current working hypothesis accommodates their dual function (both immunodulatory and PRR) through MAMP recognition, as they would; (*i*) prevent autoimmunity by dampening activation of autoreactive lymphocytes; and (*ii*) optimize the antimicrobial T cell response by favoring the activation of lymphocytes with the highest reactivity to microbial antigens [15].

**Figure 1:**
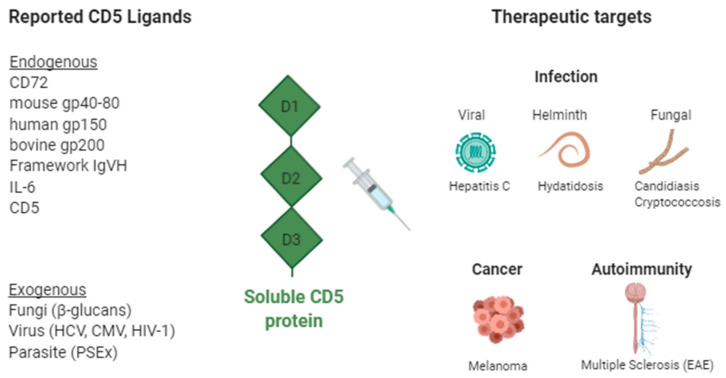
Soluble CD5 decoy receptor-based therapeutic strategies in infection, autoimmunity, and cancer. Reported CD5 ligands and their therapeutic targets. CD, cluster of differentiation; gp, glycoprotein; IL, Interleukin; IgVH, immunoglobulin heavy-chain variable; HCV, hepatitis C virus; CMV, cytomegalovirus; HIV, human immunodeficiency virus; Created with BioRender.com.

**Figure 2:**
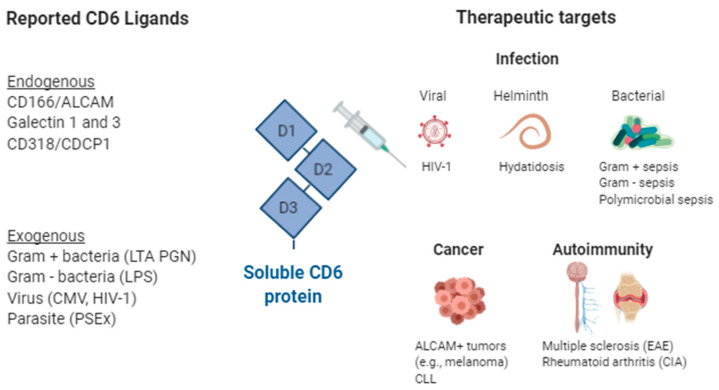
Soluble CD6 decoy receptor-based therapeutic strategies in infection, immunomodulation, autoimmunity, and cancer. Reported CD6 ligands and their therapeutic targets. ALCAM, Activated Leukocyte Cell Adhesion Molecule; CD, cluster of differentiation; CDCP1, CUB domain-containing protein 1; CIA, Collagen Induced Arthritis; CLL, chronic lymphocytic leukemia; CMV, cytomegalovirus; HIV, human immunodeficiency virus; LTA, Lipoteichoic acid; LPS, lipopolysaccharide; PGN, Peptidoglycan; Created with BioRender.com.

CD5 and CD6 were among the first lymphocyte surface receptors discovered in the 1970s with the advent of monoclonal antibody (mAb) technology [16], but their functions remained elusive until the first knockout (^−/−^) mice became available several decades later [17,18,19]. The information derived from CD5- and CD6-deficient mice has enabled a better understanding of their physiological role and brought light to their relevance and therapeutic potential in immune-related disorders. Given the ethical concerns of genetically manipulating human material, alternative approaches are necessary to transfer their pharmacologic properties into the clinic. The use of soluble CD5 and CD6 proteins as decoy receptors would enable transient functional knockdown of receptor-ligand interactions that can be readily explored in animal models of disease and further translated to patients. The following sections summarize the information available on the development of soluble CD5 and CD6 receptor-based therapeutic strategies in immune-related disorders (Figure 1 and Figure 2).

## 2. The Lymphocyte Receptor CD5

CD5 is a lymphoid-specific receptor expressed by all T cells [20,21] and certain B cell subsets involved in production of natural poly-reactive antibodies (B1a cells) and regulation of immune responses (Breg or B10 cells) [22,23]. Low surface CD5 levels have also been reported in certain macrophage [24,25], endothelial [26], and dendritic [27,28,29] cell subpopulations. The highest surface levels of CD5 are found in mouse regulatory T (Treg; CD4^+^CD25^+^Foxp3^+^) and B cells (Breg or B10 cells; CD1d^+^CD5^+^), two IL-10-producing subsets involved in the prevention of autoimmunity [23,30]. As stated above, CD5 is a signaling co-receptor, associated with the antigen-specific clonotypic receptor complex of T and B cells, and down-modulates the activation/differentiation signals delivered upon specific antigen recognition. This is achieved through the phosphorylation of Tyr, Ser and Thr residues in the cytoplasmic tail of CD5 and further interaction with downstream intracellular signal transducers. The signaling pathway used by CD5 is only partially known and we remit the reader to comprehensive reviews on that specific issue [31,32,33,34,35]. An interesting, though intriguing, finding is the demonstration that ligation of the extracellular domain of CD5 is not required for the negative regulation of TCR signaling during intra-thymic T-cell development [36].

In addition to membrane-bound CD5, the presence of a circulating soluble form of CD5 (sCD5) has been shown in sera from healthy individuals at pico/nanomolar range resulting from proteolytic cleavage following lymphocyte activation [37]. Moreover, increased levels of sCD5 have been reported in sera from patients undergoing certain autoimmune and inflammatory disorders, such as Sjögren syndrome (SS), rheumatoid arthritis, (RA) systemic inflammatory response syndrome (SIRS), and atopic dermatitis (AD), in which lymphocyte activation occurs [38,39,40,41]. It is currently unknown whether shCD5 release, following lymphocyte activation in such disorders corresponds to an epiphenomenon with no further consequences, given the scarcity of the release, of if alternatively it represents a feed-back loop in which shCD5 competes in endogenous ligand binding with membrane-bound CD5. The latter model would hinder its negative modulatory function leading to increased lymphocyte activation or even apoptosis via activation-induced cell death.

The nature of the endogenous CD5 ligand/s still remains controversial as none of the proposed candidates have been validated by independent research groups. The list of reported CD5 ligands includes: CD72 [42], IgV_H_ framework region [43], gp200 [44], gp40–80 [45,46], gp150 [47], IL-6 [48,49] and the CD5 itself [50]. Recent evidence shows that CD5 also interacts with different MAMPs (Figure 1). The extracellular region of CD5 has been reported to bind to β-glucans—a constitutive and broadly distributed component of fungal cell walls—with similar affinity (*K_d_*) to that reported for Dectin-1—the main β-glucan receptor in mammalian myeloid cells [12,51]. Such interaction is specific since no significant binding to other fungal-derived (mannan) or bacterial-derived (lipopolysaccharide, LPS; lipoteichoic acid, LTA; peptidoglycan, PGN) MAMPs was observed [12]. Importantly, exposure of HEK 293 or 2G5 Jurkat cell transfectants expressing membrane-bound CD5 to the β-glucan-rich fungal particle zymosan induced intracellular signaling events (i.e., mitogen-activated protein kinase phosphorylation) and cytokine release (i.e., IL-8) conditioned to CD5′s cytoplasmic tail integrity [12]. MAMPs recognition by CD5 has been further extended to parasitic structures from the cestode *Echinococcus granulosus* [11,52], viral structures from the Hepatitis C Virus (HCV) [53], Citomegalovirus (CMV) and Human Immunodeficiency Virus type 1 (HIV-1) (our unpublished observations).

### 2.1. Soluble CD5 as Therapeutic Agent in Infection

Following the discovery of CD5 as a β-glucan PRR, CD5-deficient (*cd5*^−/−^) mice demonstrated higher susceptibility to systemic fungal (i.e., *Candida albicans* and *Cryptococus neoformans*) infection concomitant with lower pro-inflammatory cytokine production and T and B cell activation [54]. These results highlight that CD5 is a non-redundant β-glucan receptor and an integral component of antifungal defense, thus, opening the possibility of its use in life-threatening invasive mycoses. The first proof of concept was in the demonstration of increased survival following prophylactic infusion of recombinant soluble human CD5 (rshCD5) to mice undergoing zymosan-induced generalized inflammation (ZIGI) [12]. Such prophylactically infused mice also showed decreased toxicity score, peritoneal leukocyte infiltration, pro-inflammatory cytokine production, and liver myeloperoxidase activity [12]. Recent studies have further shown dose- and time-dependent therapeutic effects of rshCD5 infusion in mouse models of systemic infection by pathogenic fungal species (*C. albicans* and *C. neoformans*) [55]. Higher rshCD5-induced survival of lethally infected mice was concomitant with reduced fungal load, increased IFN-γ mRNA levels and increased lymphoid (NK and B) and myeloid (dendritic, macrophage and granulocyte) cell infiltration in primary target organs (i.e., kidney for *C. albicans*) [55]. Furthermore, ex vivo studies showed that addition of rshCD5 to fungus-splenocyte co-cultures increased pro-inflammatory cytokine release involved in antifungal defense (TNF-α and IFN-γ) and reduced the number of viable *C. albicans.* Of note was the observation of additive mouse survival effects when rshCD5 was combined with sub-optimal doses of fluconazole. These results prompt further exploration of shCD5 as adjunctive immunotherapy to reduce the adverse effects associated with current anti-mycotic drugs without compromising their efficacy in case of drug resistance, and at the same time, to expand their antifungal spectrum. The lack of shCD5 efficacy, observed when treating *C. albicans*-infected immunodeficient NOD scid gamma (NSG) mice, supports the notion that an intact immune system is necessary for optimal survival following shCD5 infusion [55].

The therapeutic value of rshCD5 has also been studied in in vitro and in vivo experimental models of parasite infection by *E. granulosus*, responsible of human hydatidosis, also named secondary cystic equinococcosis [11]. In vitro binding studies showed that rshCD5 interacts with intact viable protoscoleces (PSC) from *E*. *granulosus* through recognition of some components from a metaperiodate-resistant fraction of tegumental antigens (PSEx), the ultimate nature of which is currently under investigation [52]. Interestingly, it was observed that (*i*) production of echinoccocal infection-protective pro-inflammatory cytokines (i.e., TNF-α and IL-6) following PSEx exposure of mouse wild-type (WT) peritoneal cells was increased in the presence of rshCD5, and (*ii*) peritoneal cells from *cd5*^−/−^ mice challenged with PSEx produced lower amounts of IL-6 compared with WT counterparts [11]. In light of such findings, rshCD5 was prophylactically infused in an experimental secondary cystic echinococcosis mouse model. The resulting reduction of infected mice, and of total count and wet weight of hydatid cysts per mouse, provides novel evidence for the prophylactic potential of rshCD5 in human cystic echinococcosis, and warrants further exploration in other helminth-driven disorders as well [11].

The role of sCD5 in murine models of viral infection has not been tested yet, but its interaction with viral structures has been reported (see above). CD5 has been identified as essential for infection of T cells with native, patient-derived HCV, enabling a lymphoid reservoir [53]. Both, antibody-mediated CD5 blockade and CD5 silencing with specific short hairpin RNA (shRNA), decreased T cell susceptibility to HCV infection in vitro [53]. These findings position sCD5 as a feasible therapeutic agent provided that sCD5-HCV interaction interferes with binding of HCV to other receptors known to be involved in hepatocyte entry [56]. Besides CD5 and HCV interaction, the possibility that sCD5 could have a role in modulating other viral infections exists as CD5 adapts its surface expression in lymphocyte subsets during Hepatitis B virus [57], HIV-1 [58], Equine Infectious Anemia [59] and Epstein-Barr Virus-associated hemophagocytic lymphohistiocytosis [60] infections.

### 2.2. Soluble CD5 as Therapeutic Agent in Cancer

The negative immunomodulatory properties of CD5 appear relevant in cancer immune response as supported by multiple evidences. Therefore, in situ adaptation (down-regulation) of CD5 expression in tumor-infiltrating lymphocytes has been shown to elicit strong anti-tumor reactivity in lung carcinoma patients [61]. Similarly, carriage of the less suppressive *CD5* haplotype Pro224-Ala471 has been associated with better survival in melanoma patients [62]. On the other hand, higher *CD5* expression is associated with better prognosis in non-small cell lung cancer, probably due to increased resistance to activation-induced cell death (AICD) of high-affinity tumor-specific T lymphocytes [63]. CD5 is also expressed on Breg cells, which play a controversial role in cancer. Therefore, Breg cells have been identified as one of the suppressive cell subtypes recruited into pancreatic ductal adenocarcinoma [64]. Circulating CD5^+^ B cells were decreased in patients with bladder cancer, probably due to either infiltration of these cells into the tumor or to the effect of T cells or cytokines [65]. Also, the presence of CD5^+^ B cells in tumor-draining lymph nodes correlated with lower staging in head and neck squamous cell carcinoma patients [66].

Studies in *cd5*^−/−^ mice have shown an increased T cell response against mouse cancer (melanoma) cells, in line with CD5′s immunomodulatory function [67]. However, this enhanced response only slowed tumor growth at early stages, since AICD phenomena prevailed at more advanced ones. Similarly, ex vivo treatment with a blocking anti-CD5 (clone 53–7.3) mAb increased the killing capacity of CD8^+^ T lymphocytes of mouse cancer (breast) cells concomitant with increased expression of markers for both T cell activation (i.e., CD69) and AICD (i.e., Fas, FasL) [68]. The latter indicates that targeting additional T cell molecules in combination with CD5 blockade may be necessary to prevent CD8^+^ T cell exhaustion and sustain CD8^+^ T cell function.

In order to assess the in vivo use of sCD5 as a decoy receptor in cancer therapy, a transgenic mouse line that constitutively expresses the soluble portion of human CD5 (shCD5) under control of the non-tissue specific SV40 promoter and immunoglobulin μ heavy chain enhancer (Eμ) was developed [69]. In these mice, serum concentrations of shCD5 were in the range of 10–100 nM and major B and T cell compartments were normal, though percentage changes were observed in some minor lymphocyte subsets: Decreased spleen transitional 1 and 2 B cells and increased spleen marginal zone B cells; decreased peritoneal and spleen Breg cells; decreased lymph node Treg cells; and increased spleen NKT cells. Similar phenotypes were observed after repeated (every-other-day) injections of purified rshCD5 for two weeks [69]. The functional relevance of such lymphocyte phenotype was supported by the demonstration of slower melanoma tumor growth in transgenic mice compared with WT controls [69]. Similarly, rshCD5 treatment (*i.p.*) in combination with chemotherapy (doxorubicin plus vincristine) of WT mice, implanted with melanoma cells, decreased tumor growth compared with mice treated with chemotherapy alone [69]. These results highlight the potential of soluble CD5 as a treatment in cancer.

Validation and mechanistic studies were further performed in a similar transgenic mouse line expressing shCD5 under the control of lymphoid-specific *lck* promoter and the Eμ enhancer to ensure preferential expression in lymphoid tissues [70]. A challenge of such transgenic mice with different tumor (melanoma and thymoma) cell lines again led to slower tumor growth compared with WT controls. Analysis of tumor-draining lymph nodes (TdLN) from transgenic mice showed higher cellularity at the expense of both CD4^+^ and CD8^+^ T cells, but with a lower percentage of Treg cells [70]. Intra-tumor mRNA analyses showed reduced IL-6 and increased IL-15 expression levels, which are known to inhibit and potentiate NK function, respectively [71,72]. The possibility that NK cells could be involved in the anti-tumor effect of transgenic shCD5 was confirmed by its reversion following treatment with the NK cell-depleting anti-NK1.1 antibody [70]. Importantly, all the above mentioned findings in transgenic mice could be reproduced in tumor-challenged WT mice administered with exogenous rshCD5 protein [70]. Moreover, ex vivo assays showed that rshCD5 interfered with polarization of naïve CD4^+^ T lymphocytes from WT mice to Treg, while favored polarization to Th1 [70]. Taken together, these studies position shCD5 administration as a potential therapeutic strategy in cancer. These treatments would work as immune checkpoint inhibitors, but enhancing anti-tumor immune responses, and by shifting immune populations towards a more pro-inflammatory status (increased effector T and NK and decreased regulatory populations).

CD5 is aberrantly expressed in B cell chronic lymphocytic leukemia (B-CLL) and mantle cell lymphoma (MCL), while other B cell malignancies, including hairy cell leukemia (HCL) and B cell prolymphocytic leukemia (B-PLL), Diffuse Large B-cell Lymphoma (DLBCL), Follicular Lymphoma (FL), Splenic marginal zone lymphoma (SMZL), and even B-Acute Lymphoblastic Leukemia (B-ALL), are usually CD5 negative or weakly positive [73]. It is thought that CD5 expression plays an important role in the development and progression of CD5^+^ B-cell malignancies due to CD5′s intracellular signaling capabilities, which regulate BCR-induced signaling and expression of several genes (e.g., IL-10) [74]. Whether blockade of intracellular signals triggered by ill-defined endogenous CD5 ligands may modify the fate of malignant B cells is an unresolved question.

### 2.3. Soluble CD5 as Therapeutic Agent in Autoimmunity

The interest of targeting CD5 in autoimmune disorders came first from mAb-mediated T cell suppression therapies. This is exemplified by the observation that treatment with the mouse IgG_1_ anti-rat CD5 OX-19 mAb [75] protected rats from T cell-dependent diabetes models [76,77], and reduced proteinuria and mesangial injury in a rat glomerulonephritis model [78]. These changes correlated with a decrease in circulating T lymphocytes, in line with autoreactive T cell depletion. However, treatment with OX-19 was also reported to increase relapse in an experimental allergic neuritis model concomitant with down-regulation of CD5 expression on T lymphocytes [79]. This may relate to CD5 high expression on Treg and Breg cells, necessary for acquisition of their suppressive function [80,81]. Therefore, anti-CD5 mAbs may have a dual effect, by inducing both internalization of membrane-bound CD5 and T cell depletion, and their balance lead to increased or decreased autoimmunity.

The use of a checkpoint inhibitor therapies has the downside of excessive immune activation, which can lead to immune-related adverse events (autoimmunity) [82]. Given the negative immunomodulatory role of the CD5 receptor in lymphocyte activation, therapies targeting CD5 would also be expected to have similar potential adverse effects. Nevertheless, studies in *cd5*^−/−^ mice report delayed onset and decreased severity of experimental autoimmune encephalomyelitis (EAE) [83]. The resistance to EAE in *cd5*^−/−^ mice was not attributed to the inability of T cells to respond efficiently to stimulation with MOG^35–55^ but was associated with elevated frequency of apoptotic activated T cells (AICD) [83]. In light of this evidence, the use of sCD5 proteins as a decoy receptor has been explored in autoimmunity models, pursuing attenuation of inflammation. Thus, administration of chimeric human CD5-Fc (hCD5-Fc) protein abrogated the formation of granular immunoglobulin deposits in peripheral capillaries and reduced production of anti-rabbit antibodies In a T-cell dependent antibody-mediated membranous glomerulonephritis (MGN) mouse model [45]. Given that putative CD5 ligands are expressed on T and B cells, the authors hypothesized that the effect was based on interference of T-B cell co-stimulation [45]. In another work, adenoviral expression of chimeric mouse CD5-Fc (mCD5-Fc) protein arrested the development of EAE, while hCD5-Fc did not [83]. Treatment with mCD5-Fc also correlated with a low activated T cell count, as a result of increased AICD. The latter suggests that blocking CD5 interactions with endogenous ligands induces apoptosis of hyper-activated autoreactive T cells. In contrast, transgenic mice expressing shCD5 develop more severe forms of EAE and collagen-induced arthritis (CIA) [69]. Several experimental differences may account for the contradicting results (e.g., use of WT vs. transgenic mice, human versus mouse protein, Fc-based vs. non-chimeric soluble proteins, etc.). In addition to the differing amounts of circulating protein (and consequently, of functional CD5 blockade) achieved by the varied expression systems used (e.g., 10–100 ng/mL shCD5 in transgenic mice vs. 1700 ng/mL mCD5-Fc in adenovirally-transduced mice) [69,83]. Consequently, the extent of CD5-ligand interference may sufficiently activate T lymphocytes and induce AICD in autoimmune settings.

## 3. The Lymphocyte Receptor CD6

CD6 is a transmembrane glycoprotein expressed on all T cells and the B1a subset of B cells [84], though it is also found in a subset of Natural Killer cells (CD56^dim^CD16^+^) [85], some hematopoietic cell precursors and certain brain cells [86,87]. As for CD5, CD6 also works as a signaling co-receptor negatively modulating the intracellular signals delivered by the antigen-specific clonotypic receptor complex (TCR and BCR) to which it is physically associated [88]. Again, this is achieved through phosphorylation of cytoplasmic Tyr, Ser and Thr residues which further interact with still ill-defined downstream intracellular signal transducers (see [89] and [90] for more extensive information on CD6 signaling).

CD6 also exist in a circulating soluble form (sCD6) at low concentrations (pM-nM) in serum of healthy individuals, and at higher levels in patients with inflammatory disorders (i.e., SS and SIRS) [38,39]. This sCD6 results from proteolytic cleavage of the membrane-bound form during lymphocyte activation events, rendering CD6^low/neg^ cells less proliferative and more sensitive to apoptosis [91].

The better characterized endogenous CD6 ligand is CD166/ALCAM (for activated leukocyte cell adhesion molecule), a member of the Ig family of adhesion molecules that is expressed on professional antigen presenting cells (APCs), but also activated lymphocytes, thymic epithelial cells, endothelial cells and brain cells [92]. The adhesive CD6-CD166/ALCAM interactions play a relevant role in the stabilization of the cell-to-cell contacts during immunological synapse formation and maturation and in the subsequent T cell proliferative responses [8,93]. Additionally reported endogenous CD6 ligands, include (*i*) CD318/CDCP1 (for CUB domain-containing protein 1) a receptor expressed on non-hematopoietic lineages, such as fibroblasts, keratinocytes, epithelial cells, and a variety of neoplastic cells [18] and (*ii*) Galectins 1 and 3, two soluble mammalian lectins [94]. While the consequences of the CD6-CD318/CDCP1 interaction still need further exploration, that of CD6 with Galectins 1 and 3 first, competes with both T cell adhesive contacts mediated by the CD6-CD166/ALCAM pairing and CD6 recognition of bacterial MAMPs (see next section below) and second, prevents Galectin 1- and 3-induced T cell apoptosis events [94].

In addition to these endogenous ligands, CD6 also binds to different MAMPs of bacterial, parasitic and viral origin (Figure 2). Indeed, CD6 binds to and senses the presence of both LPS from Gram-negative bacteria, and LTA and PGN from Gram-positive bacteria, and induces intracellular signaling events and cytokine release conditioned to CD6′s cytoplasmic tail integrity [13,14]. Interestingly, the affinity (*K_d_*) of the CD6 interaction with LPS, LTA and PGN is high [13,14] and in a range of that reported for CD14, the main macrophage receptor for such bacterial components [95]. Moreover, MAMPs recognition by CD6 also includes tegumental components (PSEx) from the cestode parasite *E. granulosus* [11,52], and glycoproteins from the HIV-1 (gp120) [96] and CMV (our unpublished observations) viruses.

### 3.1. Soluble CD6 as Therapeutic Agent in Infection

The first evidence for the in vivo therapeutic benefit of the MAMP-binding properties of CD6 in infection was the demonstration of increased survival, concomitant with reduced serum levels of pro-inflammatory cytokines (i.e., TNF-α, IL-6, and IL-1β), following prophylactic infusion of recombinant soluble human CD6 (rshCD6) to mice undergoing a lethal LPS-induced septic shock, an experimental model of severe Gram-negative infection [13]. Similar prophylactic effects of rshCD6 were later observed when mice were subjected to lethal models of septic shock induced by intraperitoneal injection of Gram-positive bacteria-derived endotoxins (i.e., LTA and PGN) or exotoxins (i.e., staphylococcal TSST-1, for toxic shock syndrome toxin 1), as well as of multidrug-resistant or -sensitive *Staphylococcus aureus* [14]. At that time, similar in vivo benefits of soluble proteins were reported for sCD14 in bacterial septic shock [97], but not in any of the SRCR superfamily of receptors.

Further studies addressed the broad-spectrum antibacterial properties of rshCD6 by challenging mice with a lethal model of polymicrobial peritonitis induced by cecal ligation and puncture (CLP model), which is considered the gold-standard pre-clinical model, resembling the progression and characteristics of human sepsis [98]. Using this model, rshCD6 infusion showed dose- and time-dependent prophylactic and therapeutic effects [99]. Protection from CLP-induced lethality was also observed in mice, expressing high and sustained serum levels (5–10 μg/mL) of mouse sCD6 (msCD6), as a result of their transduction with hepatotropic adeno-associated virus (AAV) [99]. Moreover, additive effects were observed when rshCD6 was combined with the broad-spectrum bactericidal antibiotic imipenem/cilastatin, a member of the carbapenem class that acts by inhibiting cell wall synthesis of various Gram-negative and -positive bacteria [99].

Recently, three short CD6-derived peptides have been identified and studied for its bacterial-recognition properties [100]. These sequences come from each of the three SRCR extracellular domains of CD6 and are homologous to the 11-mer consensus peptide identified in DMBT-1/SAG, which show bacterial binding properties [101]. Those CD6-derived peptides showed similar capabilities, though differing in their magnitude, regarding interaction with LPS and LTA, bacterial agglutination, and prevention of CLP-induced septic shock [100]. Consequently, these peptides offer cost-effective opportunities for developing new adjunctive alternatives to currently available sepsis treatment. One such possibility could be their covalent coupling to a solid-phase for designing adsorption devices to remove circulating bacterial toxins [102].

As for CD5, targeting CD6 in certain viral infections could represent a new therapeutic approach, though further exploration is still needed. Indeed, it has been reported that rshCD6 interferes with HIV-1 infection of human peripheral blood mononuclear cells (PBMCs) as well as with gp120 binding to human PBMC in a dose-dependent manner [96]. This is achieved through CD6 interaction with a linear sequence of the V3 loop from HIV-1 gp120, which is relevant for chemokine receptor interaction [96]. The shCD6-gp120 interaction is enhanced by pre-binding of sCD4 to gp120, suggesting that inhibitory activity of rshCD6 is mediated by blocking the gp120/coreceptor interaction [96]. These results suggest that the anti-HIV-1 activity of shCD6 likely takes place at the virological synapse, thus preventing HIV-1 entry.

With respect to parasites, rshCD6 directly interacts with tegumental antigens (PSEx) from *E. granulosus* and down-modulate the IL-10, TNF-α and IL-6 cytokine responses of mouse peritoneal cells exposed to them [11]. Accordingly, rshCD6 infusion has exhibited some degree of prophylactic potential in a mouse model of secondary cystic echinococcosis, by showing a trend towards reduction in the proportion of infected mice and the number of hydatid cysts per mouse [11].

### 3.2. Soluble CD6 as Therapeutic Agent in Cancer

The ability of CD6 to modulate important physiological lymphocyte processes warrants exploration of its immunomodulatory potential in cancer therapy. Indeed, CD6 expression was early reported in chronic lymphocytic leukemia (CLL) and in some lymphosarcoma cell leukemia (LSCL) cells [84], despite CD6 expression did not correlate with disease progression [103]. However, it is known that CD6 ligation induces expression of anti-apoptotic proteins and prevents apoptosis of leukemic B cells following IgM cross-linking [104]. Therefore, the prevention of CD6 ligation on leukemic cells increases their sensitivity to apoptosis and limit their abnormal expansion.

The CD6 ligand CD166/ALCAM is involved in the maintenance of tissue architecture, immune responses and tumor progression [105]. CD166/ALCAM establishes both homophilic (ALCAM-ALCAM) and heterophilic (ALCAM-CD6) cell-to-cell interactions, with the former being around 100-fold weaker than the heterophilic ones [93]. Heterophilic ALCAM-CD6 interactions are involved in lymphocyte migration and extravasation processes [106]. A number of studies support the association of CD166/ALCAM expression with aggressiveness in a variety of cancers, including melanoma, prostate, breast, ovarian, esophageal, bladder and intestinal cancers (probably a result of homophilic ALCAM-ALCAM interactions), thus constituting of an oncology-related target and prognostic marker [107,108].

The use of sCD6 has been explored in different experimental instances as an alternative to blocking anti-CD166/ALCAM mAbs. In vitro studies showed that rshCD6 inhibits T-cell proliferation to an extent comparable with CD6-blocking mAbs or chimeric ALCAM-Fc proteins suggesting, at least in part, to be the result of interfering heterophilic ALCAM-CD6 interactions (e.g., APC–T-cell interactions) [8,109]. Furthermore, rshCD6 inhibits proliferation and migration of tumor cell lines expressing high CD166/ALCAM surface levels (B16-F0, EL-4, and MC-205) [110].

Recent work provides proof-of-concept on the immunotherapeutic potential of sCD6 in cancer and its translatability to the clinical practice [110]. This was explored by challenging genetically-modified or WT mice, expressing high circulating levels of sCD6 with subcutaneous or metastatic syngeneic cancer cells of different lineage origins (B16-F0 melanoma, MCA-205 sarcoma and RMA-S lymphoma cells). The results showed delayed in vivo growth of tumor cells constitutively expressing high CD166/ALCAM surface levels in transgenic shCD6*lck*EμTg mice compared with WT controls [110]. Moreover, a lower number of lung metastases and improved survival was observed when WT mice transduced with hepatotropic AAV expressing soluble mouse CD6 (AAV-smCD6) were challenged (*i.v.*) with B16.F0 melanoma cells. Importantly, both delayed local growth and lower metastatization results were observed in tumor-challenged WT mice infused with rshCD6 protein [110]. In vitro studies showed that mechanisms operating at the level of lymphocyte effector function and tumorigenicity were engaged in the presence of rshCD6, such as defective Treg generation and function, decreased CD166/ALCAM-mediated tumor cell proliferation/migration and impaired galectin-induced T-cell apoptosis [110]. These pre-clinical results illustrate the multifaceted effects of sCD6 on cancer development and support its immunotherapeutic potential, as well its translatability to clinical oncotherapy.

### 3.3. Soluble CD6 as Therapeutic Agent in Autoimmunity

Growing evidence backs up the use of sCD6 for the treatment of autoimmune disorders. Single nucleotide polymorphisms (SNPs) of *CD6* have been associated with susceptibility and/or clinical outcome of several autoimmune diseases, including multiple sclerosis (MS), RA, (SS, inflammatory bowel disease (IBD), Behcet’s disease and psoriasis [88,111]. Intriguingly, lower mRNA *CD6* levels have been reported in PBMC from MS patients, without any significant association with *CD6* SNPs [112]. Though apparently contradictory, relevant information also comes from *cd6*^−/−^ mice, in which attenuated or aggravated forms of experimental autoimmune diseases have been reported depending on the disease model (EAE, autoimmune uveitis, graft versus host disease-induced lupus-like, imiquimod-induced psoriasis, CIA) and the mouse genetic background (C57BL/6, DBA-1) [19,99,113,114,115,116,117]. Some of the referred *cd6^−/−^* mice results might relate in part to the fact that the CD6-CD166/ALCAM interaction has been shown to be important in the recruitment of peripheral blood leukocytes to inflamed tissues [106,118].

In this context and despite the lack of a thorough understanding of CD6 function, availability of mouse and humanized anti-CD6 mAbs has provided valuable information regarding the potential targeting of CD6 for the treatment of RA, psoriasis and potentially other T cell–driven autoimmune conditions [119]. Indeed, ALZUMAb^®^ (Itolizumab), a humanized anti-human CD6 mAb developed from its parent murine antibody IOR-T1, has been approved by the Drugs Controller General of India in January 2013 to treat psoriasis [120,121]. A randomized phase III clinical trial was carried out in India in a cohort of 225 patients with moderate to severe chronic psoriasis plaques in which Itolizumab was effective and well-tolerated [122].

In the light of the above, the use of sCD6 could provide an effective alternative. Indeed, preliminary observations made with shCD6*lck*EμTg mice and rshCD6-treated WT mice reveal improved outcomes (lower clinical score) in two different experimental autoimmune diseases (CIA and EAE) [110] and warrant further clinical validation.

## 4. Concluding Remarks

The multifaceted abilities of the CD5 and CD6 receptors set the basis for potential breakthrough therapies. CD5 and CD6 combine immunomodulatory and PRR properties, and are suited to treating pathologies associated to dysregulated immune responses. The fact that soluble forms of these lymphoid scavenger receptors interact with different endogenous and exogenous ligands (some still awaiting full description), has prompted incipient pre-clinical studies in infectious, autoimmune and cancerous processes. Exogenous sCD5 and sCD6 proteins offer efficient and safe therapeutic agents, and their testing in a clinical setting has now become a priority.

## References

[1] Martinez V.G., Moestrup S.K., Holmskov U., Mollenhauer J., Lozano F. (2011). The conserved scavenger receptor cysteine-rich superfamily in therapy and diagnosis. Pharmacol. Rev..

[2] Pombinho R., Sousa S., Cabanes D. (2018). Scavenger Receptors: Promiscuous Players during Microbial Pathogenesis. Crit. Rev. Microbiol..

[3] PrabhuDas M.R., Baldwin C.L., Bollyky P.L., Bowdish D.M.E., Drickamer K., Febbraio M., Herz J., Kobzik L., Krieger M., Loike J. (2017). A Consensus Definitive Classification of Scavenger Receptors and Their Roles in Health and Disease. J. Immunol..

[4] Sarrias M.R., Grønlund J., Padilla O., Madsen J., Holmskov U., Lozano F. (2004). The Scavenger Receptor Cysteine-Rich (SRCR) domain: An ancient and highly conserved protein module of the innate immune system. Crit. Rev. Immunol..

[5] Gimferrer I., Farnós M., Calvo M., Mittelbrunn M., Enrich C., Sánchez-Madrid F., Vives J., Lozano F. (2003). The accessory molecules CD5 and CD6 associate on the membrane of lymphoid T cells. J. Biol. Chem..

[6] Beyers A.D., Spruyt L.L., Williams A.F. (1992). Multimolecular associations of the T-cell antigen receptor. Trends Cell Biol..

[7] Lankester A.C., van Schijndel G.M., Cordell J.L., van Noesel C.J., van Lier R.A. (1994). CD5 is associated with the human B cell antigen receptor complex. Eur. J. Immunol..

[8] Gimferrer I., Calvo M., Mittelbrunn M., Farnós M., Sarrias M.R., Enrich C., Vives J., Sánchez-Madrid F., Lozano F. (2004). Relevance of CD6-Mediated Interactions in T Cell Activation and Proliferation. J. Immunol..

[9] Cho J.H., Sprent J. (2018). TCR tuning of T cell subsets. Immunol. Rev..

[10] Alizadeh M., Safarzadeh A., Hoseini S.A., Piryaei R., Mansoori B., Hajiasgharzadeh K., Baghbanzadeh A., Baradaran B. (2020). The potentials of immune checkpoints for the treatment of blood malignancies. Crit. Rev. Oncol. Hematol..

[11] Mourglia-Ettlin G., Miles S., Velasco-De-Andrés M., Armiger-Borràs N., Cucher M., Dematteis S., Lozano F. (2018). The ectodomains of the lymphocyte scavenger receptors CD5 and CD6 interact with tegumental antigens from Echinococcus granulosus sensu lato and protect mice against secondary cystic echinococcosis. PLoS Negl. Trop. Dis..

[12] Vera J., Fenutria R., Canadas O., Figueras M., Mota R., Sarrias M.-R., Williams D.L., Casals C., Yelamos J., Lozano F. (2009). The CD5 ectodomain interacts with conserved fungal cell wall components and protects from zymosan-induced septic shock-like syndrome. Proc. Natl. Acad. Sci. USA.

[13] Sarrias M.-R., Farnos M., Mota R., Sanchez-Barbero F., Ibanez A., Gimferrer I., Vera J., Fenutria R., Casals C., Yelamos J. (2007). CD6 binds to pathogen-associated molecular patterns and protects from LPS-induced septic shock. Proc. Natl. Acad. Sci. USA.

[14] Martínez-Florensa M., Consuegra-Fernández M., Martínez V.G., Cañadas O., Armiger-Borràs N., Bonet-Roselló L., Farrán A., Vila J., Casals C., Lozano F. (2014). Targeting of key pathogenic factors from gram-positive bacteria by the soluble ectodomain of the scavenger-like lymphocyte receptor CD6. J. Infect. Dis..

[15] Lenz L.L. (2009). CD5 sweetens lymphocyte responses. Proc. Natl. Acad. Sci. USA.

[16] Bernard A., Boumsell L., Dausset J., Milstein C., Schlossman S.F., Bernard A., Boumsell L., Dausset J., Milstein C., Schlossman S.F. (1984). Leucocyte Typing.

[17] Tarakhovsky A., Kanner S.B., Hombach J., Ledbetter J.A., Müller W., Killeen N., Rajewsky K. (1995). A role for CD5 in TCR-mediated signal transduction and thymocyte selection. Science.

[18] Enyindah-Asonye G., Li Y., Ruth J.H., Spassov D.S., Hebron K.E., Zijlstra A., Moasser M.M., Wang B., Singer N.G., Cui H. (2017). CD318 is a ligand for CD6. Proc. Natl. Acad. Sci. USA.

[19] Orta-Mascaró M., Consuegra-Fernández M., Carreras E., Roncagalli R., Carreras-Sureda A., Alvarez P., Girard L., Simões I., Martínez-Florensa M., Aranda F. (2016). CD6 modulates thymocyte selection and peripheral T cell homeostasis. J. Exp. Med..

[20] Reinherz E.L., Kung P.C., Goldstein G., Schlossman S.F. (1979). A monoclonal antibody with selective reactivity with functionally mature human thymocytes and all peripheral human T cells. J. Immunol..

[21] Ledbetter J.A., Rouse R.V., Micklem H.S., Herzenberg L.A. (1980). T cell subsets defined by expression of Lyt-1,2,3 and Thy-1 antigens. Two-parameter immunofluorescence and cytotoxicity analysis with monoclonal antibodies modifies current views. J. Exp. Med..

[22] Berland R., Wortis H.H. (2002). Origins and functions of B-1 cells with notes on the role of CD5. Annu. Rev. Immunol.

[23] Yanaba K., Bouaziz J.-D., Matsushita T., Tsubata T., Tedder T.F. (2009). The Development and Function of Regulatory B Cells Expressing IL-10 (B10 Cells) Requires Antigen Receptor Diversity and TLR Signals. J. Immunol..

[24] Borrello M.A., Palis J., Phipps R.P. (2001). The relationship of CD5+ B lymphocytes to macrophages: Insights from normal biphenotypic B/macrophage cells. Int. Rev. Immunol..

[25] Moreau M.F., Thibaud J.L., Miled L.B., Chaussepied M., Baumgartner M., Davis W.C., Minoprio P., Langsley G. (1999). Theileria annulata in CD5(+) macrophages and B1 B cells. Infect. Immun..

[26] Gogolin-Ewens K., Meeusen E., Lee C.-S., Brandon M. (1989). Expression of CD5, a lymphocyte surface antigen on the endothelium of blood vessels. Eur. J. Immunol..

[27] De Bernardis F., Lucciarini R., Boccanera M., Amantini C., Arancia S., Morrone S., Mosca M., Cassone A., Santoni G. (2006). Phenotypic and Functional Characterization of Vaginal Dendritic Cells in a Rat Model of Candida albicans Vaginitis. Infect. Immun..

[28] Korenfeld D., Gorvel L., Munk A., Man J., Schaffer A., Tung T., Mann C., Klechevsky E. (2017). A type of human skin dendritic cell marked by CD5 is associated with the development of inflammatory skin disease. JCI Insight.

[29] Li H., Burgueño-Bucio E., Xu S., Das S., Olguin-Alor R., Elmets C.A., Athar M., Raman C., Soldevila G., Xu H. (2019). CD5 on dendritic cells regulates CD4+ and CD8+ T cell activation and induction of immune responses. PLoS ONE.

[30] Ordoñez-Rueda D., Lozano F., Sarukhan A., Raman C., Garcia-Zepeda E.A., Soldevila G. (2009). Increased numbers of thymic and peripheral CD4 ^+^ CD25 ^+^ Foxp3 ^+^ cells in the absence of CD5 signaling. Eur. J. Immunol..

[31] Lozano F., Simarro M., Calvo J., Vila J.M., Padilla O., Bowen M.A., Campbell K.S. (2000). CD5 signal transduction: Positive or negative modulation of antigen receptor signaling. Crit. Rev. Immunol..

[32] Soldevila G., Raman C., Lozano F. (2011). The immunomodulatory properties of the CD5 lymphocyte receptor in health and disease. Curr. Opin. Immunol..

[33] Freitas C., Johnson D., Weber K. (2018). T Cell Calcium Signaling Regulation by the Co-Receptor CD5. Int. J. Mol. Sci..

[34] Voisinne G., Gonzalez de Peredo A., Roncagalli R. (2018). CD5, an Undercover Regulator of TCR Signaling. Front. Immunol..

[35] Burgueño-Bucio E., Mier-Aguilar C.A., Soldevila G. (2019). The multiple faces of CD5. J. Leukoc. Biol..

[36] Bhandoola A., Sambandam A., Allman D., Meraz A., Schwarz B. (2003). Early T Lineage Progenitors: New Insights, but Old Questions Remain. J. Immunol..

[37] Calvo J., Places L., Espinosa G., Padilla O., Vilà J.M., Villamor N., Ingelmo M., Gallart T., Vives J., Font J. (1999). Identification of a natural soluble form of human CD5. Tissue Antigens.

[38] Ramos-Casals M., Font J., García-Carrasco M., Calvo J., Places L., Padilla O., Cervera R., Bowen M.A., Lozano F., Ingelmo M. (2001). High circulating levels of soluble scavenger receptors (sCD5 and sCD6) in patients with primary Sjögren’s syndrome. Rheumatology.

[39] Aibar J., Martínez-Florensa M., Castro P., Carrasco E., Escoda-Ferran C., Fernández S., Butjosa M., Hernández C., Rinaudo M., Lozano F. (2015). Pattern of soluble CD5 and CD6 lymphocyte receptors in critically ill patients with septic syndromes. J. Crit. Care.

[40] Jamin C., Magadur G., Lamour A., Mackenzie L., Lydyard P., Katsikis P., Youinou P. (1992). Cell-free CD5 in patients with rheumatic diseases. Immunol. Lett..

[41] Noh G.W., Lee K.Y. (1998). Circulating Soluble CD5 in Atopic Dermatitis. Mol. Cells.

[42] Van de Velde H., von Hoegen I., Luo W., Parnes J.R., Thielemans K. (1991). The B-cell surface protein CD72/Lyb-2 is the ligand for CDS. Nature.

[43] Pospisil R., Silverman G.J., Marti G.E., Aruffo A., Bowen M.A., Mage R.G. (2000). CD5 is A potential selecting ligand for B-cell surface immunoglobulin: A possible role in maintenance and selective expansion of normal and malignant B cells. Leuk. Lymphoma.

[44] Haas K.M., Estes D.M. (2001). The identification and characterization of a ligand for bovine CD5. J. Immunol..

[45] Biancone L., Bowen M.A., Lim A., Aruffo A., Andres G., Stamenkovic I. (1996). Identification of a novel inducible cell-surface ligand of CD5 on activated lymphocytes. J. Exp. Med..

[46] Bikah G., Carey J., Ciallella J.R., Tarakhovsky A., Bondada S. (1996). CD5-mediated negative regulation of antigen receptor-induced growth signals in B-1 B cells. Science.

[47] Calvo J., Places L., Padilla O., Vilà J.M., Vives J., Bowen M.A., Lozano F. (1999). Interaction of recombinant and natural soluble CD5 forms with an alternative cell surface ligand. Eur. J. Immunol..

[48] Masuda K., Kishimoto T. (2016). CD5: A New Partner for IL-6. Immunity.

[49] Zhang C., Xin H., Zhang W., Yazaki P.J., Zhang Z., Le K., Li W., Lee H., Kwak L., Forman S. (2016). CD5 Binds to Interleukin-6 and Induces a Feed-Forward Loop with the Transcription Factor STAT3 in B Cells to Promote Cancer. Immunity.

[50] Brown M.H., Lacey E. (2010). A Ligand for CD5 Is CD5. J. Immunol..

[51] Adams E.L., Rice P.J., Graves B., Ensley H.E., Yu H., Brown G.D., Gordon S., Monteiro M.A., Papp-Szabo E., Lowman D.W. (2008). Differential high-affinity interaction of dectin-1 with natural or synthetic glucans is dependent upon primary structure and is influenced by polymer chain length and side-chain branching. J. Pharmacol. Exp. Ther..

[52] Miles S., Velasco-de-Andrés M., Lozano F., Mourglia-Ettlin G. (2020). Interactome analysis of CD5 and CD6 ectodomains with tegumental antigens from the helminth parasite Echinococcus granulosus sensu lato. Int. J. Biol. Macromol..

[53] Sarhan M.A., Pham T.N.Q., Chen A.Y., Michalak T.I. (2012). Hepatitis C virus infection of human T lymphocytes is mediated by CD5. J. Virol..

[54] Velasco-de-Andrés M., Català C., Casadó-Llombart S., Simões I., Zaragoza O., Carreras E., Lozano F. (2020). The lymphocyte scavenger receptor CD5 plays a nonredundant role in fungal infection. Cell. Mol. Immunol..

[55] Velasco-de Andrés M., Català C., Casadó-Llombart S., Martínez-Florensa M., Simões I., García-Luna J., Mourglia-Ettlin G., Zaragoza Ó., Carreras E., Lozano F. (2020). The lymphocytic scavenger receptor CD5 shows therapeutic potential in mouse models of fungal infection. Antimicrob. Agents Chemother..

[56] Sarhan M.A., Chen A.Y., Michalak T.I. (2013). Differential Expression of Candidate Virus Receptors in Human T Lymphocytes Prone or Resistant to Infection with Patient-Derived Hepatitis C Virus. PLoS ONE.

[57] Sun H., Lv J., Tu Z., Hu X., Yan H., Pan Y., Xu D., Lian Z., Chi X., Niu J. (2013). Antiviral treatment improves disrupted peripheral B lymphocyte homeostasis in chronic hepatitis B virus-infected patients. Exp. Biol. Med..

[58] Penney S.J., Gallant M.E., Grant M.D. (2014). Greater frequency of CD5-negative CD8+ T cells against human immunodeficiency virus type 1 than other viruses is consistent with adaptation to antigenic variation. AIDS Res. Ther..

[59] Tumas D.B., Hines M.T., Perryman L.E., Davis W.C., McGuire T.C. (1994). Corticosteroid Immunosuppression and Monoclonal Antibody-mediated CD5+ T Lymphocyte Depletion in Normal and Equine Infectious Anaemia Virus-carrier Horses. J. Gen. Virol..

[60] Karandikar N.J., Kroft S.H., Yegappan S., Rogers B.B., Aquino V.M., Lee K.-M., Kumar V., Guenaga F.J., Jaffe E.S., Douek D.C. (2004). Unusual immunophenotype of CD8+ T cells in familial hemophagocytic lymphohistiocytosis. Blood.

[61] Dorothée G., Vergnon I., El Hage F., Chansac B.L.M., Ferrand V., Lécluse Y., Opolon P., Chouaib S., Bismuth G., Mami-Chouaib F. (2005). In Situ Sensory Adaptation of Tumor-Infiltrating T Lymphocytes to Peptide-MHC Levels Elicits Strong Antitumor Reactivity. J. Immunol..

[62] Potrony M., Carreras E., Aranda F., Zimmer L., Puig-Butille J.-A., Tell-Martí G., Armiger N., Sucker A., Giménez-Xavier P., Martínez-Florensa M. (2016). Inherited functional variants of the lymphocyte receptor CD5 influence melanoma survival. Int. J. Cancer.

[63] Moreno-Manuel A., Jantus-Lewintre E., Simões I., Aranda F., Calabuig-Fariñas S., Carreras E., Zúñiga S., Saenger Y., Rosell R., Camps C. (2020). CD5 and CD6 as immunoregulatory biomarkers in non-small cell lung cancer. Transl. Lung Cancer Res..

[64] Das S., Shapiro B., Vucic E.A., Vogt S., Bar-Sagi D. (2020). Tumor cell-derived IL1β promotes desmoplasia and immune suppression in pancreatic cancer. Cancer Res..

[65] Roudafshani Z., Jazayeri M.H., Mahmoudi A.R., Nedaeinia R., Safari E., Jazayeri A. (2019). Evaluation of the frequency of CD5+ B cells as natural immunoglobulin M producers and circulating soluble CD5 in patients with bladder cancer. Mol. Biol. Rep..

[66] Norouzian M., Mehdipour F., Balouchi Anaraki S., Ashraf M.J., Khademi B., Ghaderi A. (2019). Atypical Memory and Regulatory B Cell Subsets in Tumor Draining Lymph Nodes of Head and Neck Squamous Cell Carcinoma Correlate with Good Prognostic Factors. Head Neck Pathol..

[67] Tabbekh M., Franciszkiewicz K., Haouas H., Lécluse Y., Benihoud K., Raman C., Mami-Chouaib F. (2011). Rescue of Tumor-Infiltrating Lymphocytes from Activation-Induced Cell Death Enhances the Antitumor CTL Response in CD5-Deficient Mice. J. Immunol..

[68] Alotaibi F., Rytelewski M., Figueredo R., Zareardalan R., Zhang M., Ferguson P.J., Maleki Vareki S., Najajreh Y., El-Hajjar M., Zheng X. (2020). CD5 blockade enhances ex vivo CD8+ T cell activation and tumour cell cytotoxicity. Eur. J. Immunol..

[69] Fenutría R., Martinez V.G., Simões I., Postigo J., Gil V., Martínez-Florensa M., Sintes J., Naves R., Cashman K.S., Alberola-Ila J. (2014). Transgenic expression of soluble human CD5 enhances experimentally-induced autoimmune and anti-tumoral immune responses. PLoS ONE.

[70] Simões I.T., Aranda F., Carreras E., Andrés M.V., Casadó-Llombart S., Martinez V.G., Lozano F. (2017). Immunomodulatory effects of soluble CD5 on experimental tumor models. Oncotarget.

[71] Cifaldi L., Prencipe G., Caiello I., Bracaglia C., Locatelli F., De Benedetti F., Strippoli R. (2015). Inhibition of Natural Killer Cell Cytotoxicity by Interleukin-6: Implications for the Pathogenesis of Macrophage Activation Syndrome. Arthritis Rheumatol..

[72] Rautela J., Huntington N.D. (2017). IL-15 signaling in NK cell cancer immunotherapy. Curr. Opin. Immunol..

[73] Jaseb K., Purrahman D., Shahrabi S., Ghanavat M., Rezaeean H., Saki N. (2019). Prognostic significance of aberrant CD5 expression in B-cell leukemia. Oncol. Rev..

[74] Gary-Gouy H., Sainz-Perez A., Marteau J.-B., Marfaing-Koka A., Delic J., Merle-Beral H., Galanaud P., Dalloul A. (2007). Natural Phosphorylation of CD5 in Chronic Lymphocytic Leukemia B Cells and Analysis of CD5-Regulated Genes in a B Cell Line Suggest a Role for CD5 in Malignant Phenotype. J. Immunol..

[75] Dallman M.J., Thomas M.L., Green J.R. (1984). MRC OX-19: A monoclonal antibody that labels rat T lymphocytes and augments in vitro proliferative responses. Eur. J. Immunol..

[76] Like A.A., Biron C.A., Weringer E.J., Byman K., Sroczynski E., Guberski D.L. (1986). Prevention of diabetes in biobreeding/worcester rats with monoclonal antibodies that recognize T lymphocytes or natural killer cells. J. Exp. Med..

[77] Ellerman K.E., Richards C.A., Guberski D.L., Shek W.R., Like A.A. (1996). Kilham rat virus triggers T-cell-dependent autoimmune diabetes in multiple strains of rat. Diabetes.

[78] Ikezumi Y., Kawachi H., Toyabe S., Uchiyama M., Shimizu F. (2000). An anti-CD5 monoclonal antibody ameliorates proteinuria and glomerular lesions in rat mesangioproliferative glomerulonephritis. Kidney Int..

[79] Strigård K., Olsson T., Larsson P., Holmdahl R., Klareskog L. (1988). Modulation of experimental allergic neuritis in rats by in vivo treatment with monoclonal anti T cell antibodies. J. Neurol. Sci..

[80] Gary-Gouy H., Harriague J., Bismuth G., Platzer C., Schmitt C., Dalloul A.H. (2002). Human CD5 promotes B-cell survival through stimulation of autocrine IL-10 production. Blood.

[81] Blaize G., Daniels-Treffandier H., Aloulou M., Rouquié N., Yang C., Marcellin M., Gador M., Benamar M., Ducatez M., Song K.D. (2020). CD5 signalosome coordinates antagonist TCR signals to control the generation of Treg cells induced by foreign antigens. Proc. Natl. Acad. Sci. USA.

[82] Bajwa R., Cheema A., Khan T., Amirpour A., Paul A., Chaughtai S., Patel S., Patel T., Bramson J., Gupta V. (2019). Adverse Effects of Immune Checkpoint Inhibitors (Programmed Death-1 Inhibitors and Cytotoxic T-Lymphocyte-Associated Protein-4 Inhibitors): Results of a Retrospective Study. J. Clin. Med. Res..

[83] Axtell R.C., Webb M.S., Barnum S.R., Raman C. (2004). Cutting Edge: Critical Role for CD5 in Experimental Autoimmune Encephalomyelitis: Inhibition of Engagement Reverses Disease in Mice. J. Immunol..

[84] Kamoun M., Kadin M.E., Martin P.J., Nettleton J., Hansen J.A. (1981). A novel human T cell antigen preferentially expressed on mature T cells and shared by both well and poorly differentiated B cell leukemias and lymphomas. J. Immunol..

[85] Braun M., Müller B., Ter Meer D., Raffegerst S., Simm B., Wilde S., Spranger S., Ellwart J., Mosetter B., Umansky L. (2011). The CD6 scavenger receptor is differentially expressed on a CD56 dim natural killer cell subpopulation and contributes to natural killer-derived cytokine and chemokine secretion. J. Innate Immun..

[86] Cortés F., Deschaseaux F., Uchida N., Labastie M.C., Friera A.M., He D., Charbord P., Péault B. (1999). HCA, an immunoglobulin-like adhesion molecule present on the earliest human hematopoietic precursor cells, is also expressed by stromal cells in blood-forming tissues. Blood.

[87] Konno A., Ahn J.S., Kitamura H., Hamilton M.J., Gebe J.A., Aruffo A., Davis W.C. (2001). Tissue distribution of CD6 and CD6 ligand in cattle: Expression of the CD6 ligand (CD166) in the autonomic nervous system of cattle and the human. J. Leukoc. Biol..

[88] Consuegra-Fernández M., Lin F., Fox D.A., Lozano F. (2018). Clinical and experimental evidence for targeting CD6 in immune-based disorders. Autoimmun. Rev..

[89] Gonçalves C.M., Henriques S.N., Santos R.F., Carmo A.M. (2018). CD6, a rheostat-type signalosome that tunes T cell activation. Front. Immunol..

[90] Mori D., Grégoire C., Voisinne G., Celis-Gutierrez J., Aussel R., Girard L., Camus M., Marcellin M., Argenty J., Burlet-Schiltz O. (2021). The T cell CD6 receptor operates a multitask signalosome with opposite functions in T cell activation. J. Exp. Med..

[91] Carrasco E., Escoda-Ferran C., Climent N., Miró-Julià C., Simões I.T., Martínez-Florensa M., Sarukhan A., Carreras E., Lozano F. (2017). Human CD6 Down-Modulation following T-Cell Activation Compromises Lymphocyte Survival and Proliferative Responses. Front. Immunol..

[92] Bowen M.A., Patel D.D., Li X., Modrell B., Malacko A.R., Wang W.C., Marquardt H., Neubauer M., Pesando J.M., Francke U. (1995). Cloning, mapping, and characterization of activated leukocyte-cell adhesion molecule (ALCAM), a CD6 ligand. J. Exp. Med..

[93] Hassan N.J., Barclay A.N., Brown M.H. (2004). Frontline: Optimal T cell activation requires the engagement of CD6 and CD166. Eur. J. Immunol..

[94] Escoda-Ferran C., Carrasco E., Caballero-Baños M., Miró-Julià C., Martínez-Florensa M., Consuegra-Fernández M., Martínez V.G., Liu F.-T., Lozano F. (2014). Modulation of CD6 function through interaction with Galectin-1 and -3. FEBS Lett..

[95] Tobias P.S., Soldau K., Gegner J.A., Mintz D., Ulevitch R.J. (1995). Lipopolysaccharide binding protein-mediated complexation of lipopolysaccharide with soluble CD14. J. Biol. Chem..

[96] Carrasco E., Escoda C., Alvarez-Fernández C., Sanchez-Palomino S., Carreras E., Gatell J.M., Gallart T., García F., Climent N., Lozano F. (2014). A Role for Scavenger-like Lymphocyte Receptor CD6 in HIV-1 Viral Infection. AIDS Res. Hum. Retrovir..

[97] Jacque B., Stephan K., Smirnova I., Kim B., Gilling D., Poltorak A. (2006). Mice expressing high levels of soluble CD14 retain LPS in the circulation and are resistant to LPS-induced lethality. Eur. J. Immunol..

[98] Dejager L., Pinheiro I., Dejonckheere E., Libert C. (2011). Cecal ligation and puncture: The gold standard model for polymicrobial sepsis?. Trends Microbiol..

[99] Martínez-Florensa M., Consuegra-Fernández M., Aranda F., Armiger-Borràs N., Di Scala M., Carrasco E., Pachón J., Vila J., González-Aseguinolaza G., Lozano F. (2017). Protective Effects of Human and Mouse Soluble Scavenger-Like CD6 Lymphocyte Receptor in a Lethal Model of Polymicrobial Sepsis. Antimicrob. Agents Chemother..

[100] Martínez-Florensa M., Català C., Velasco-de Andrés M., Cañadas O., Fraile-Ágreda V., Casadó-Llombart S., Armiger-Borràs N., Consuegra-Fernández M., Casals C., Lozano F. (2018). Conserved Bacterial-Binding Peptides of the Scavenger-Like Human Lymphocyte Receptor CD6 Protect From Mouse Experimental Sepsis. Front. Immunol..

[101] Bikker F.J., Ligtenberg A.J.M., End C., Renner M., Blaich S., Lyer S., Wittig R., van’t Hof W., Veerman E.C.I., Nazmi K. (2004). Bacteria Binding by DMBT1/SAG/gp-340 Is Confined to the VEVL *XXXX* W Motif in Its Scavenger Receptor Cysteine-rich Domains. J. Biol. Chem..

[102] Zimmermann M., Busch K., Kuhn S., Zeppezauer M. (1999). Endotoxin adsorbent based on immobilized human serum albumin. Clin. Chem. Lab. Med..

[103] Sembries S., Pahl H., Stilgenbauer S., Döhner H., Schriever F. (1999). Reduced expression of adhesion molecules and cell signaling receptors by chronic lymphocytic leukemia cells with 11q deletion. Blood.

[104] Osorio L.M., Jondal M., Aguilar-Santelises M. (1998). Regulation of B-CLL apoptosis through membrane receptors and Bcl-2 family proteins. Leuk. Lymphoma.

[105] Swart G.W.M. (2002). Activated leukocyte cell adhesion molecule (CD166/ALCAM): Developmental and mechanistic aspects of cell clustering and cell migration. Eur. J. Cell Biol..

[106] Cayrol R., Wosik K., Berard J.L., Dodelet-Devillers A., Ifergan I., Kebir H., Haqqani A.S., Kreymborg K., Krug S., Moumdjian R. (2008). Activated leukocyte cell adhesion molecule promotes leukocyte trafficking into the central nervous system. Nat. Immunol..

[107] Weidle U.H., Eggle D., Klostermann S., Swart G.W.M. (2010). ALCAM/CD166: Cancer-related issues. Cancer Genom. Proteom..

[108] Darvishi B., Boroumandieh S., Majidzadeh-A K., Salehi M., Jafari F., Farahmand L. (2020). The role of activated leukocyte cell adhesion molecule (ALCAM) in cancer progression, invasion, metastasis and recurrence: A novel cancer stem cell marker and tumor-specific prognostic marker. Exp. Mol. Pathol..

[109] Kim Y.S., Kim M.N., Lee K.E., Hong J.Y., Oh M.S., Kim S.Y., Kim K.W., Sohn M.H. (2018). Activated leucocyte cell adhesion molecule (ALCAM/CD166) regulates T cell responses in a murine model of food allergy. Clin. Exp. Immunol..

[110] Simões I.T., Aranda F., Casadó-Llombart S., Velasco-de Andrés M., Català C., Álvarez P., Consuegra-Fernández M., Orta-Mascaró M., Merino R., Merino J. (2020). Multifaceted effects of soluble human CD6 in experimental cancer models. J. Immunother. Cancer.

[111] Kofler D.M., Farkas A., von Bergwelt-Baildon M., Hafler D.A. (2016). The Link Between CD6 and Autoimmunity: Genetic and Cellular Associations. Curr. Drug Targets.

[112] Wagner M., Bilinska M., Pokryszko-Dragan A., Sobczynski M., Cyrul M., Kusnierczyk P., Jasek M. (2014). ALCAM and CD6-multiple sclerosis risk factors. J. Neuroimmunol..

[113] Li Y., Singer N.G., Whitbred J., Bowen M.A., Fox D.A., Lin F. (2017). CD6 as a potential target for treating multiple sclerosis. Proc. Natl. Acad. Sci. USA.

[114] Consuegra-Fernández M., Martínez-Florensa M., Aranda F., de Salort J., Armiger-Borràs N., Lozano T., Casares N., Lasarte J.J., Engel P., Lozano F. (2017). Relevance of CD6-mediated interactions in the regulation of peripheral T-cell responses and tolerance. Front. Immunol..

[115] Consuegra-Fernández M., Julià M., Martínez-Florensa M., Aranda F., Català C., Armiger-Borràs N., Arias M.T., Santiago F., Guilabert A., Esteve A. (2018). Genetic and experimental evidence for the involvement of the CD6 lymphocyte receptor in psoriasis. Cell. Mol. Immunol..

[116] Zhang L., Li Y., Qiu W., Bell B.A., Dvorina N., Baldwin W.M., Singer N., Kern T., Caspi R.R., Fox D.A. (2018). Targeting CD6 for the treatment of experimental autoimmune uveitis. J. Autoimmun..

[117] Li Y., Ruth J.H., Rasmussen S.M., Athukorala K.S., Weber D.P., Amin M.A., Campbell P.L., Singer N.G., Fox D.A., Lin F. (2020). Attenuation of Murine Collagen-Induced Arthritis by Targeting CD6. Arthritis Rheumatol..

[118] Levesque M.C., Heinly C.S., Whichard L.P., Patel D.D. (1998). Cytokine-regulated expression of activated leukocyte cell adhesion molecule (CD166) on monocyte-lineage cells and in rheumatoid arthritis synovium. Arthritis Rheum..

[119] Hernández P., Moreno E., Aira L.E., Rodríguez P.C. (2016). Therapeutic Targeting of CD6 in Autoimmune Diseases: A Review of Cuban Clinical Studies with the Antibodies IOR-T1 and Itolizumab. Curr. Drug Targets.

[120] Jayaraman K. (2013). Biocon’s first-in-class anti-CD6 mAb reaches the market. Nat. Biotechnol..

[121] Dogra S., Shabeer D., Rajagopalan M. (2020). Anti-CD6 mAbs for the treatment of psoriasis. Expert Opin. Biol. Ther..

[122] Krupashankar D.S., Dogra S., Kura M., Saraswat A., Budamakuntla L., Sumathy T.K., Shah R., Gopal M.G., Narayana Rao T., Srinivas C.R. (2014). Efficacy and safety of itolizumab, a novel anti-CD6 monoclonal antibody, in patients with moderate to severe chronic plaque psoriasis: Results of a double-blind, randomized, placebo-controlled, phase-III study. J. Am. Acad. Dermatol..

